# The Brain Overwork Scale: A Population-Based Cross-Sectional Study on the Psychometric Properties of a New 10-Item Scale to Assess Mental Distress in Mongolia

**DOI:** 10.3390/healthcare11071003

**Published:** 2023-03-31

**Authors:** Battuvshin Lkhagvasuren, Tetsuya Hiramoto, Enkhnaran Tumurbaatar, Enkhjin Bat-Erdene, Gantsetseg Tumur-Ochir, Vijay Viswanath, Joshua Corrigan, Tsolmon Jadamba

**Affiliations:** 1 Brain Science Institute, Mongolian National University of Medical Sciences, Ulaanbaatar 14210, Mongolia; 2 Brain and Mind Research Institute, Mongolian Academy of Sciences, Ulaanbaatar 16066, Mongolia; 3 Department of Psychosomatic Medicine, International University of Health and Welfare Narita Hospital, Chiba 286-0124, Japan; 4 Department of Psychosomatic Medicine, Fukuoka National Hospital, National Hospital Organization, Fukuoka 811-1394, Japan; 5 Child Health Institute of New Jersey, Department of Neuroscience and Cell Biology, Robert Wood Johnson Medical School, Rutgers University, New Brunswick, NJ 08901, USA; 6 Department of Mental Health Surveillance, National Center for Mental Health, Ulaanbaatar 13280, Mongolia; 7 College of Medicine, University of Cincinnati, Cincinnati, OH 45267, USA; 8 Department of Biological Engineering, Massachusetts Institute of Technology, Cambridge, MA 02139, USA

**Keywords:** health psychology, mental distress, stress, population, psychometric property, validation, Mongolia

## Abstract

Identifying mental distress is a complex task, particularly when individuals experience physical symptoms. Traditional self-report questionnaires that detect psychiatric symptoms using emotional words may not work for these individuals. Consequently, there is a need for a screening tool that can identify both the physical and mental symptoms of mental distress in individuals without a clinical diagnosis. Our study aimed to develop and validate a scale that measures mental distress by measuring the extent of brain overwork, which can be extrapolated as the burden of mental distress. In this population-based cross-sectional study, we recruited a total of 739 adults aged 16–65 years from 64 sampling centers of a cohort in Mongolia to validate a 10-item self-report questionnaire. Internal consistency was measured using McDonald’s ω coefficient. Test–retest reliability was analyzed using intraclass correlation coefficients. Construct and convergent validities were examined using principal component analysis (PCA) and confirmatory factor analysis (CFA). The Hospital Anxiety and Depression Scale (HADS) and the abbreviated version of World Health Organization Quality of Life (WHOQOL-BREF) were used to evaluate criterion validity. Among the participants, 70.9% were women, 22% held a bachelor’s degree or higher, 38.8% were employed, and 66% were married. The overall McDonald’s ω coefficient was 0.861, demonstrating evidence of excellent internal consistency. The total intraclass correlation coefficient of the test–retest analysis was 0.75, indicating moderate external reliability. PCA and CFA established a three-domain structure that provided an excellent fit to the data (RMSEA = 0.033, TLI = 0.984, CFI = 0.989, χ^2^ = 58, *p* = 0.003). This 10-item scale, the Brain Overwork Scale (BOS-10), determines mental distress in three dimensions: excessive thinking, hypersensitivity, and restless behavior. All the items had higher item-total correlations with their corresponding domain than they did with the other domains, and correlations between the domain scores had a range of 0.547–0.615. BOS-10 correlated with HADS, whereas it was inversely correlated with WHOQOL-BREF. In conclusion, the results suggest that BOS-10 is a valid and reliable instrument for assessing mental distress in the general population. The scale screens for mental distress that is characterized by subjective symptoms such as excessive thinking, hypersensitivity, and restless behavior. The current findings also demonstrate that the BOS-10 is quantitative, simple, and applicable for large group testing. This scale may be useful for identifying at-risk individuals who may require further evaluation and treatment for mental distress.

## 1. Introduction

The number of entities that determine the diagnosis of a disease or an abnormal condition is nearly 55,000 in the tenth revision of the International Classification of Diseases (ICD-10), and it rose up to 85,000 in its current revision (ICD-11) [1]. In accordance with recent advances in medicine, life expectancy, quality of life, and public health have improved across the globe. Public awareness of mental health is increasing gradually. However, the prevalence of mental disorders has significantly increased in recent decades worldwide [2,3]. One in four people in the world are affected by a mental disorder [4]. Furthermore, one in five people who are not diagnosed with psychiatric or neurological disorders may have mental distress or mental health problems. Mental distress decreases the quality of life, causes disability, and increases mortality [5]. Therefore, mental distress is an important public health burden, and there is a need for its early detection and intervention.

Self-report questionnaires are widely used tools to screen mental distress in individuals. To identify the gaps in knowledge of screening mental distress, we reviewed widely used questionnaires that measure different aspects of mental distress, such as Generalized Anxiety Disorder-7 (GAD-7), Beck Depression Inventory (BDI), Hospital Anxiety and Depression Scale (HADS) [6], Patient Health Questionnaire-9 (PHQ-9), Kessler Psychological Distress (K10), and Perceived Stress Scale (PSS). GAD-7 assesses the severity of anxiety symptoms such as restlessness, worrying, and difficulty concentrating [7]. However, GAD-7 does not assess physical symptoms such as muscle tension, headaches, or gastrointestinal concerns. BDI assesses the severity of depressive symptoms including feelings of sadness, guilt, and loss of pleasure in activities [8]. This scale has good psychometric properties, and its reliability and validity have been extensively studied in various populations. However, similarly, BDI does not screen for physical symptoms such as pain, fatigue, or changes in appetite or sleep. K10 assesses non-specific mental distress symptoms such as nervousness, hopelessness, and worthlessness [9,10]. PSS measures the degree to which an individual perceives their life as unpredictable, uncontrollable, and overloaded [11]. However, K10 and PSS both do not assess physical symptoms such as pain, fatigue, or changes in appetite or sleep. Overall, the aforementioned questionnaires have strong psychometric properties with a focus on emotional symptoms. However, mental distress causes not only emotional, but also physical and behavioral symptoms.

Some individuals with mental distress may only present with physical symptoms, while they deny any mental symptoms [12]. These individuals are typically referred to non-psychiatric wards and may be diagnosed with conditions, such as medically unexplained symptoms and functional somatic syndromes, or conditions with unknown origins, as conventional medical tests and examinations do not explain their subjective symptoms [13,14]. However, these patients often respond positively to treatments with antidepressants and anxiolytics, indicating that the etiopathology of mental distress may be associated with chronic stress-induced dysfunctions of brain activity [15,16,17]. As a result, questionnaires that rely only on emotional words to detect psychiatric symptoms may not be effective for identifying these individuals.

To identify self-report questionnaires that screen for both emotional and physical symptoms of mental distress, we conducted a brief bibliometric analysis using the Scopus database, searching for articles published between 1970 and 2022 using a search query with “self-report questionnaires” and “mental distress screening” as the main keywords. This search resulted in a total of 10,473 publications in the field of self-report questionnaires for mental distress screening between 1970 and 2022, with an average of 239 publications per year. Although this brief analysis did not use advanced approaches, such as multi-criteria analysis, P-median model, or Promethee-Gaia method [18,19,20], the analysis revealed a gap in the literature regarding the screening of physical and behavioral symptoms in self-report questionnaires for mental distress, highlighting the need for more research in this area. Furthermore, some people deny any emotional or behavioral symptoms or do not readily express their emotional state in questionnaires. Particularly, people with alexithymia do not easily recognize their mental distress [21]. Alexithymia, a trait characterized by difficulties in identifying, describing, and expressing emotions, is measured by tools such as Toronto Alexithymia Scale-20 (TAS-20). TAS-20 is a useful scale for screening emotional difficulties; however, it is important to recognize its limitations in screening for physical and behavioral symptoms beyond simply emotional symptoms [22,23,24,25].

Taken together, identifying mental distress is often complicated, and there is no screening tool that particularly identifies both physical and mental symptoms of mental distress in people without a clinical diagnosis. Therefore, there is an urgent need for additional screening tools that address physical symptoms of mental distress to provide a comprehensive evaluation of an individual’s mental health. These tools could help in the early detection and treatment of mental distress, thereby improving the overall quality of life of individuals experiencing mental distress.

Since Selye postulated his stress theory based on his findings that the nonspecific response of the body to any demand results in adrenal hyperactivity, lymphatic atrophy, and peptic ulcer (referred to as a classic triad), the effects of stress on brain functions have been well recognized. He distinguished acute stress from chronic stress or a response to chronically applied stressors, termed “general adaptation syndrome”, by introducing a basic concept of the hypothalamic–pituitary–adrenocortical axis as a main mediator that influences brain activity to maintain homeostasis in response to challenges [26]. At present, a diversity of stress mediators, in addition to the corticotropin-releasing hormone response, including, but not limited to the catecholaminergic pathway or sympathetic–adrenomedullary axis, the acetylcholinergic pathway, or parasympathetic nervous system, and a recently emerging neuroimmune pathway, have been established [27,28,29]. However, so far, a direct biomarker that determines how strongly the nervous system copes with mental distress does not exist. Thus, untangling the fundamental problem of how psychological stress can produce various mental and somatic symptoms, or even diseases, has not yet been well described. Therefore, we hypothesized that chronic stress might cause the restless overwork of brain activity. Any psychological event that induces mental distress may be associated with cognitive, emotional, and behavioral alterations. To cope with mental distress, brain activity increases, particularly via increased sympathetic nervous system activity. If this heightened activity is not corrected, it may lead to exhaustion or the overwork of brain activity, eventually resulting in mental disorders [30,31]. Aging, alexithymia, alexisomia, and other predispositions make individuals vulnerable to mental overwork and further complications leading to diseases, including both mental and physical disorders [21,32,33,34]. In accordance with our hypothesis, previous studies suggested that people who have recurrent mental symptoms have typical characteristics of neuroticism, including a symptom referred to as “thinking too much” [29,35]. We propose that mental distress can be characterized by subjective symptoms of excessive thinking, hypersensitivity, restless behavior, social withdrawal, and minor problems in daily life, which constitute an abnormal condition called brain overwork syndrome.

This study aims to develop a novel tool for assessing mental distress that could be useful for both clinicians and the public. The objectives of this study are (1) to develop a novel scale that measures brain overwork symptoms and (2) to determine the psychometric properties of this novel scale in the general population.

## 2. Materials and Methods

### 2.1. Development of a Novel Inventory for Detecting Mental Distress

Our conceptual framework aimed to outline the symptoms that describe the severity of mental distress in association with brain overwork symptoms. As shown in Figure S1, chronic psychosocial stressors cause mental distress or an abnormal condition that is characterized by excessive thinking (ET), hypersensitivity (H), restless behavior (RB), social withdrawal (SW), and minor problems in daily life (MP) [29,30,35,36,37]. These five dimensions were described by 37 items and constitute the brain overwork syndrome; thus, we called this new questionnaire the Brain Overwork Scale (BOS). To minimize self-report and social desirability biases, we developed these items with a preference for focusing on behavioral characteristics instead of emotional characteristics (mood changes). The 37 items consisted of statements of agreement measured on a 5-point Likert scale (Table S1). BOS was administered to the participants of the cohort described below. Following data collection, items that scored a factor loading lower than 0.4 on a single domain with principal component analysis (PCA) with varimax rotation were eliminated. Furthermore, items with more than 10% missing responses, which might be unintelligible to participants, were removed. The remaining 10 items constituted the final version of the BOS (BOS-10), which was further examined for its psychometric properties.

### 2.2. Study Population

This cross-sectional study was conducted as a part of a nationwide population-based cohort study that investigated brain-related disorders in the general population in Mongolia. To cover a full representative population, we included two residency locations (urban and rural), resulting in the desired sample size of 770. Considering a response rate of 80%, 924 individuals who fulfilled the inclusion criteria were invited to participate in the study. Mongolian citizens who lived in the geopolitical units for at least six months and who were not diagnosed with any disease were considered to meet the inclusion criteria. If selected participants were not available at the center for the on-site survey, they were replaced by the next available participant regardless of age, gender, social status, and employment status. The subjects were recruited from 64 sampling centers, including 30 centers of 8 districts in Ulaanbaatar and 34 centers of 4 rural regions in Mongolia.

Among the invited individuals, 9 participants did not physically show up at a sampling center. Out of 915 approached, 109 participants declined to participate, and 67 were excluded due to incomplete survey data. The remaining 739 subjects were enrolled for the final analysis (Figure S2).

### 2.3. Data Collection

The data collection began on 7 September 2020, and the preliminary dataset was extracted on 19 January 2021. The study was conducted in the official language of Mongolia (Mongolian). Based on a pilot test using a draft version of the BOS questionnaire, the expert committee reviewed the adaptation process and finalized BOS-10. All participants completed tablet-based questionnaires addressing their demographics and the BOS questionnaire. A total of 366 participants completed the BOS questionnaire again within two weeks of the initial administration to assess test–retest reliability. All field study members completed a data collection training program prior to the study.

### 2.4. Instruments

Hospital Anxiety and Depression Scale (HADS) and the abbreviated version of World Health Organization Quality of Life (WHOQOL-BREF) were used to evaluate the criterion validity of the BOS. HADS is a 14-item self-report questionnaire that is widely used to evaluate the severity of anxiety and depression symptoms that occurred in the past week [6]. Among fourteen items, seven of them are for anxiety (HADS-A subscale), and the remaining seven are for depression (HADS-D subscale). The Mongolian version of HADS demonstrated evidence of good validity and reliability for the general population [38]. Each item is rated on 4-point scale for a total score between 0 and 21 for each subscale. The ranges of scores for cases on each subscale are 0–7 (normal), 8–10 (mild abnormality), 11–14 (moderate abnormality), and 15–21 (severe abnormality).

WHOQOL-BREF was developed by the WHOQOL group in 1996 [39]. It contains 24 items, and each item is measured on a 5-point Likert scale. This questionnaire determines the quality of life (QOL) with four domains: physical QOL, psychological QOL, social relationships, and environmental QOL. The score for each domain consists of a mean score of items, for which a higher score indicates a better QOL regarding that aspect. Two additional single items measure the overall perception of QOL and general health. The Mongolian version of WHOQOL-BREF had good reliability and validity for assessing QOL in the general population in Mongolia [40].

### 2.5. Statistical Analysis

The study characteristics were expressed as means, with a standard deviation for normally distributed variables, and as numbers with percentages in cases of categorical data. The reliability of the questionnaire was assessed by McDonald’s omega (excellent ≥ 0.9; good ≥ 0.8; acceptable ≥ 0.7) for internal consistency. A test–retest procedure was performed at a two-week interval using the intraclass correlation coefficient (ICC). ICC values of <0.5, from 0.5 to 0.7, from 0.7 to 0.9, and >0.9 were considered to have poor, moderate, good, and excellent test–retest reliability, respectively [41]. Validity was evaluated by principal component analysis (PCA) with varimax rotation. Factor analysis suitability was examined by the Bartlett test of sphericity (*p* < 0.001) and Kaiser–Meye–Olkin (KMO) test of sampling adequacy (*p* > 0.65), followed by determining the number of relevant factors via eigenvalue analysis. The factors with eigenvalues > 1 were assumed to be meaningful and retained for rotation [42]. A factor loading of 0.4 was established as the lower bound for a variable to be included in the respective factor structure [41]. Confirmatory factor analysis (CFA) was performed to assess the BOS 3-factor model fit using the following criteria for the structural equation modeling: the Tucker–Lewis index (TLI), a comparative fit index (CFI) close to 0.90 or above, and the root-mean-square error of approximation (RMSEA < 0.05). To assess convergent validity, Spearman’s rank correlations were calculated with the HADS and WHOQOL-BREF. The correlation coefficients of 0.10–0.39, 0.40–0.69, 0.70–0.89, and 0.9–1.0 were considered to be weak, moderate, strong, very strong, respectively [43]. All data analyses were performed using JAMOVI v. 2.2.5, except for the ICC and CFA, which was performed using IBM SPSS v.21 and Amos v.26. Statistical significance was set at *p* < 0.05, and all tests were two-tailed ones.

### 2.6. Ethics Approval and Consent to Participate

All procedures performed in this study were performed in accordance with the ethical standards of the institutional and/or national research committee and the 1964 Helsinki Declaration and its later amendments. The design and methods were reviewed and approved by the ethics committee at the Mongolian National University of Medical Sciences, Ulaanbaatar, Mongolia (number: MNUMS 20/03-05). This study was not a trial and did not require registration. Written informed consent was obtained from all participants.

## 3. Results

### 3.1. Demographics and Response Distributions

This study comprised 739 participants aged 16–65 years, with a mean age and standard deviation of 37.42 ± 14.7 years (Table 1).

Among them, 524 (70.9%) were females, 593 (80.2%) were residents in Ulaanbaatar city, 488 (66%) were married, 411 (55.6%) graduated high school or a school below this level, 287 (38.8%) were employed, and 500 (67.7%) had a low income. The total scores of BOS-10 were different between the age groups (Kruskal–Wallis test, *p* < 0.001), whereas they did not differ between gender (Mann–Whitney test, *p* = 0.220). The unadjusted total score of BOS-10 was 20.3, whereas after age and gender adjustment, it was 21.9 and 26.3, respectively (Figure S3).

Table 2 presents the distribution of responses for each BOS item in three domains.

The response distributions were skewed toward better conditions, indicating floor effects (>29%), with the exception that there was no skew in the responses to items regarding excessive thinking. A full range of responses to all items indicated there were no ceiling effects.

### 3.2. Reliability

Item analyses included inspecting means, standard deviations, item-to-total correlations, Cronbach’s α, and McDonald’s ω to determine internal consistency (Table 3).

McDonald’s ω coefficients for the Mongolian version of the BOS questionnaire were as follows: overall BOS, 0.861; excessive thinking domain, 0.908; hypersensitivity domain, 0.909; restless behavior domain, 0.889. The overall Cronbach’s α coefficient of the BOS questionnaire was 0.859. The component scores of each item were significantly correlated with the rest of them, the range of which was 0.520–0.628.

ICC was calculated using a three-factor mixed-effects model with a 95% CI to determine external reliability (Table 4).

A test–retest study was carried out on 366 participants, with an interval of 16 ± 2.3 days between two time points. Participants were aged from 16 to 29, and the mean age was 21.55 ± 1.94; 194 (53%) were females. The results from ICC analyses showed that the total score of BOS was 0.75, which indicates good reliability [41]. ICC values of the domains were as follows: excessive thinking domain, 0.73; hypersensitivity, 0.69; restless behavior domain, 0.65.

### 3.3. Validity

**Figure 1:**
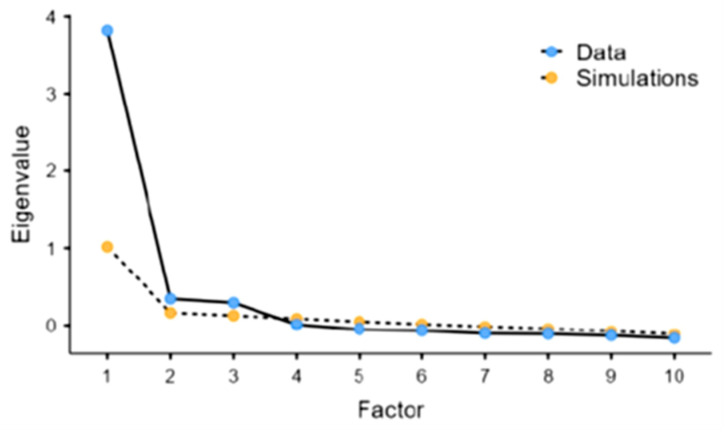
Scree plot for 10 items of the BOS (*n* = 739).

The 95% confidence intervals for the means of each item are plotted in Figure S4. PCA using the varimax rotation method for 10 items of data identified three components with an eigenvalue greater than 1, as presented by the scree plot in Figure 1.

The three components account for 22.9%, 20.3%, and 20% of the variance, resulting in 63.2% of the total variance (Table 5).

The KMO value was 0.908, and Bartlett’s test of sphericity was significant (*p* < 0.001), which indicated that the dataset was adequately sampled and that factor analysis of the data was appropriate.

**Figure 2:**
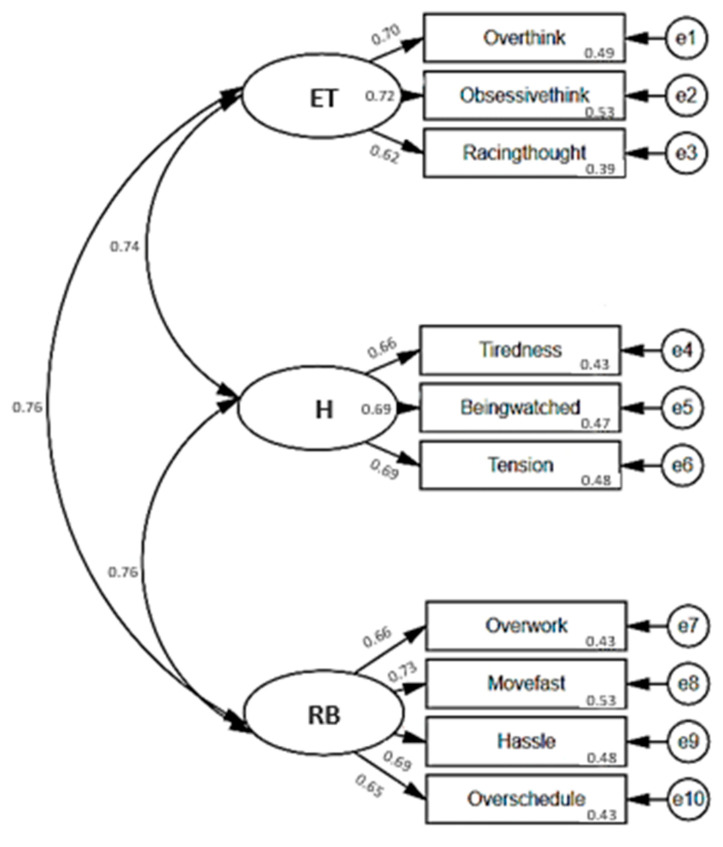
Confirmatory factor analysis path diagram for the three domains of BOS (*n* = 739). ET: Excessive thinking; H: Hypersensitivity; RB: Restless behavior.

We tested the three-component model including 10 items to evaluate how well the three domains were combined to identify the underlying construct of the BOS using CFA. Figure 2 presents the factor correlations and loading.

The model had excellent fit indices (RMSEA = 0.0332; CFI = 0.989; TLI = 0.984) and a test for exact fit showed a significant difference (χ^2^(32) = 58.1, *p* = 0.003). Ellipses represent subscales. Covariances of errors between items with similar content are shown. The arrows in Figure 2 are the factor loadings, representing direct effects of the indicators on the latent BOS. The value had ranges of 0.74–0.76 for the correlation coefficients between the domains and 0.62–0.73 for the standardized regression weights. The squared multiple correlations were 0.43–0.53, whereas the measurement errors were represented from e1 to e10.

To determine convergent validity, we analyzed the correlation of each item with its corresponding domain (corrected item–total correlations) and inter-item correlations between the domains (Table 6).

Each item correlated more strongly (≥0.7) with its corresponding domain than they did with the other domains [43]. No item correlated more strongly with another domain than it did with its corresponding domain. Therefore, 10 out of 10 items (100%) met the criterion for item convergence. These results supported the three-domain structure.

To determine criterion validity, we calculated the correlations of the BOS total score and domain scores with the HADS and WHOQOL-BREF total scores and domain scores (Table 7).

The BOS total score correlated with the HADS scores (anxiety: *r* = 0.429, *p* < 0.001; depression: *r* = 0.288, *p* < 0.001), which indicates that the BOS identifies anxiety and depression. In contrast, the total BOS scores inversely correlated with the WHOQOL-BREF domain scores (physical health: *r* = −0.396, *p* < 0.001; psychological health: *r* = −0.277, *p* < 0.001; social relations: *r* = −0.161, *p* < 0.001; environmental health: *r* = −0.323, *p* < 0.001), which suggests that the BOS indicates a poor QOL. Each BOS domain had similar results in correlation with those of HADS and WHOQOL-BREF; the only exception was that the restless behavior domain of the BOS did not correlate with the social relationship domain of WHOQOL-BREF.

## 4. Discussion

We developed a new self-assessment scale, the BOS, which is presented as a reliable instrument for screening mental distress in the general population. The results indicate that the BOS has excellent internal consistency and moderate external reliability. Explorative PCA obtained three constructs including 10 items, which were confirmed by CFA, indicating good construct validity. These three dimensions (ET, H, and RB) constituted the final version of the BOS (BOS-10), which has been demonstrated as a valid measure of the severity of mental distress in healthy subjects with no diagnosis. The results of criterion validity suggest that the BOS-10 scores estimate the severity of mental distress, particularly anxiety and depression. The BOS-10 total score and subscale scores were moderately associated with anxiety, whereas only H showed relatively strong correlation with depression. BOS-10 also depicts a decreased quality of life. The results suggest that the BOS-10 scores indicate a decrease in physical, psychological, social and environmental health, except for RB.

Out of five main dimensions that we initially hypothesized in our conceptual framework, ET, H, and RB were confirmed by PCA and CFA. ET is the core symptom of brain overwork syndrome, which reflects exhaustion of brain activity due to rumination thinking. We carefully selected items for ET based on clinical observations. Additionally, previous studies indicated that people with symptoms such as “thinking too much”, “too much thinking”, or “too much use of brain” complained of feeling isolated from the world due to their neurotic mind, indicating that brain activity was affected with neuroticism and hypersensitivity [35,37,44]. Many studies indicated that life events, childhood maltreatment, negative feedback from parents, and a family history of psychiatric diseases were related to the establishment of rumination thinking, which might be a critical pathology of depression and anxiety disorders [45,46]. Furthermore, rumination thinking has been associated with neuroticism and physical diseases, such as diabetes, arthritis, stomach/gallbladder diseases, and a chronic cough, in many clinical investigations [47,48,49,50]. Moreover, some patients also tend to pay attention to subtle thoughts that might be associated with past psychological events or future worries. Therefore, we proposed to use excessive thinking, as it covers not only rumination, but also obsessive thinking.

Although H is associated with abnormal reactions to physical stimuli such as drugs, light, or noise, we used this symptom to describe an abnormal condition in which someone becomes highly sensitive to psychological stimuli, such as communication difficulties, eye contact, or voice tone. H might be associated with mental fatigue or brain fatigue that results in mental disorders, including chronic fatigue syndrome, depressive disorders, or suicide [51,52].

RB refers to a cluster of symptoms that are commonly associated with resistance to stress, as conceptualized by the general adaptation syndrome perspective [53]. Individuals who experience excessive thinking, particularly those with high levels of anxiety, may demonstrate a tendency to move quickly and have a preoccupation with time, which can result in hurried behaviors in daily activities [54]. Psychosocial stress has been established to influence both brain activity and behavior. In the past, Canon discovered that activation of the sympathetic and adrenal medulla systems occurs when cats are frightened by barking dogs, causing them to exhibit the “fight or flight” response [55]. In vivo studies have also demonstrated that psychosocial stress results in increased marble burying behaviors, locomotion activity, and scratching behaviors [56,57,58,59]. Additionally, behavioral patterns appear to vary across different illnesses in human subjects. For instance, the manic phase of bipolar disorder and obsessive compulsive disorder tend to be associated with increased obsessive/repetitive behaviors and hyperactivity, whereas depression tends to be associated with increased suppressive behavior [36,60,61]. In this study, the RB domain was related to active behavior, and the SW domain was related to suppressive behavior. Our results led us to retain the RB domain and remove the SW domain. This finding may be attributed to the fact that this study was conducted on healthy individuals with normal activity levels, rather than on individuals with illnesses. Moreover, we observed a high correlation between the RB and ET domains, which suggests that RB may be linked to excessive brain activity. Previous research has also revealed that individuals with highly neurotic personalities tend to be more creative and play a more significant role in the advertising industry, both of which are positive aspects [62,63]. However, excessive rumination has also been identified as a risk factor for mental illness [64,65]. Therefore, when one is preventing and treating mental illness, it is crucial to consider both brain overwork and behavior patterns in patients. Despite our understanding of the impact of psychosocial stress on brain activity and behavior, numerous aspects of this relationship remain unclear. More research is necessary not only in healthy subjects, but also in individuals with illnesses, such as depressive, anxiety, obsessive compulsive, and bipolar disorders.

This new tool might be helpful for detecting mental distress in individuals who are difficult to assess with conventional questionnaires. The BOS consists of items with simple and concrete expressions that are easily recalled from diverse daily life perspectives. Therefore, it might be useful to identify patients who have alexithymia and alexisomia. Alexithymia is a personality trait characterized by difficulties with the awareness and expression of one’s own emotions [21]. Sifneos introduced the term and indicated that alexithymia is associated with psychosomatic diseases including inflammatory bowel disease and asthma [66,67]. In clinical practice, many patients complain of physical symptoms that cannot be clearly explained even with appropriate medical examinations, and these patients are usually diagnosed as having medically unexplained symptoms (MUS) or functional somatic syndrome (FSS) [68]. These patients tended to show traits of alexithymia, including difficulty with identifying emotions, describing feelings to others, and externally oriented thinking [32,34]. Previous literature has shown that patients with alexithymia express their mental distress as relatively strong physical symptoms [69]. Moreover, alexisomia is characterized by personality traits of difficulties in identifying and expressing somatic sensations, meaning that patients lack words to describe their bodily states [33,51]. Patients with alexisomia tend to have difficulties expressing their mental and somatic symptoms, making them hard to understand by clinicians. Taken together, patients with alexithymia or alexisomia are thought to have difficulties with being aware of and expressing their emotional and physical states. The BOS is composed of items that are made to be easy to understand for subjects with alexithymia and alexisomia. In clinical settings, this tool might be useful for assessing mental distress in patients with MUS and FSS.

Currently, it is not fully understood how mental distress causes psychiatric disorders. Recent in vivo studies have demonstrated that psychological stress impairs brain structures, such as the hippocampus, extended amygdala, and midbrain raphe, and leads to memory impairment, maladaptive behaviors, and vulnerability to psychosocial stress [70,71,72]. For example, repeated stress exposures decrease spinogenesis and spine stability in the dorsal CA1 pyramidal neurons of the hippocampus in mice, indicating cognitive deficits [70]. In clinical studies, oxidative stress, neuroinflammation, and maladjustment of the gut microbiota might be involved in the pathophysiology of stress-related disorders [51]. Taken together, this study provided a new tool to measure different dimensions of mental distress that are distinct from anxiety or depression, and we validated it in a nonclinical population.

As for the population features, the gender composition of the study sample did not accurately represent the gender distribution of the overall population. Women constituted 70% of the sample, deviating from the gender distribution in Mongolia. Previous studies have shown that women have a higher prevalence of mental disorders, such as depression and anxiety, compared to that of men [73,74]. However, in this study, we did not find any difference in the total score between genders. Mongolia does not share the male dominance that is seen in some other Asian cultures, and this may diminish the impact of gender differences on mental health symptoms. Additionally, it is possible that women in Mongolia may be more interested in their healthcare and are thus more willing to cooperate in sample collection for research purposes. Additionally, notable differences in the total scores of the BOS-10 were observed among different age groups in the study sample. Specifically, we found that the total score was significantly higher in the younger age group. As this instrument was designed to explore brain overwork by evaluating items related to excessive thinking and restlessness, it is plausible that higher values were obtained in highly active, young individuals. Within the younger age group, we observed higher anxiety scores and a higher total score on BOS-10. Over the past few years, the exposure of children and adolescents to digital information has increased, particularly in urban settings. This exposure may generate harmful feelings and emotions that can elicit mental and physical symptoms. Therefore, detecting excessive brain activity may be useful in screening young individuals for anxiety. Overall, age groups must be accounted for when one is conducting research utilizing the new scale. Furthermore, Mongolia is a country with economic underdevelopment, a high proportion of impoverished individuals, and a high prevalence of violence [75]. The majority of study participants belonged to the low-income bracket. However, it is important to note that the country’s relatively inexpensive cost of living, attributable to low utility and grocery prices, may have influenced the sample recruitment. Nonetheless, poverty and violence are known to exert a deleterious effect on mental health, particularly among younger individuals [76]. Therefore, researchers using the new scale must consider that BOS-10 was developed and validated in a young population with a considerable proportion of low-income participants.

This study has several methodological limitations and shortcomings. First, as participants were recruited from the general population in Mongolia, the three-dimensional structure of BOS-10 is only appropriate for the particular study population. It is recommended to use the 37-item version of BOS with five dimensions if a different population is being tested. We eliminated two dimensions, including SW and MP symptoms, from the 37-item version for BOS-10. Those dimensions were included to detect subjects who avoid asking for help from others and try to do everything themselves so as not to bother others. However, these characteristics were not typical of Mongolian adults, of whom half are cooperative, nomadic herders in the countryside. Moreover, nomadic people live close to nature and are relatively free from intensive digital media, in contrast to urban dwellers. Second, the disparity in age groups of the sample had significant impacts on the survey results. Future studies should describe the influence of socioeconomic status on their findings. We also suggest that the 37-item version of the BOS should be utilized in other regions, as mental symptoms have been shown to vary significantly across countries. Third, we did not perform a comprehensive bibliometric analysis, which could have provided additional insights into the existing literature on mental distress screening tools. While bibliometric analysis is a useful tool to explore the scientific landscape of a specific research field, we faced methodological difficulties in conducting a systematic literature review and machine learning-based analysis for the predictive assessment of screening tools in this study [77,78]. Future researchers should consider conducting bibliometric analysis to identify the current state of the field and to highlight any research gaps that need to be addressed. Fourth, although construct, convergent, and criterion validities were assessed, this study did not examine the discriminant validity. Furthermore, as a cross-sectional study, it did not provide information regarding the persistence of mental distress over time.

To improve the validity of the instrument, future studies should compare this tool with other relevant measurement inventories. Moreover, to further determine the sensitivity and specificity of the instrument, the assessment should be conducted in both clinical and nonclinical settings using a longitudinal design.

## 5. Conclusions

In conclusion, the results suggest that BOS-10 is a valid and reliable instrument for assessing mental distress in the general population of Mongolia. The scale demonstrated excellent internal consistency and moderate external reliability, supporting its utility as a tool for identifying individuals who may require further evaluation and treatment for mental distress. However, it is important to note that this study has some limitations, including the lack of advanced analysis for predictive assessment of the screening scale and comprehensive bibliometric analysis for exposing the main gaps in mental health care. Additionally, as mental symptoms can vary significantly across countries, it may be advised to utilize the 37-item version of the BOS in other regions. Despite the limitations, BOS-10 may be a useful tool for identifying individuals who may require further evaluation and treatment for mental distress. The identification of these individuals can be a crucial first step in addressing mental health concerns in the general population. Further clinical studies are needed to explain the pathophysiological mechanisms underlying brain overwork syndrome and to explore the potential benefits of using BOS-10 in clinical settings. Overall, this study contributes to our understanding of mental distress in the general population and highlights the potential for using BOS-10 as a screening tool for mental health professionals and researchers.

## Figures and Tables

**Table 1:** Demographic characteristics of the study subjects (*n* = 739).

Demographic Characteristics		Total	
		*n*	%
		739	100
Age (years)	(mean ± SD)	37.42 ± 14.7	
Age groups (years)	16–29	281	38
	30–39	128	17.3
	40–49	136	18.4
	50–59	133	18
	>60	61	8.3
Gender	Male	215	29.1
	Female	524	70.9
Residency	Ulaanbaatar city	143	19.8
	Rural areas	593	80.2
Marital status	Never married	221	29.9
	Married	488	66
	Others *	30	4.1
Education level	High school or below	411	55.6
	Below bachelor’s degree	166	22.5
	Bachelor’s degree	146	19.8
	Above bachelor’s degree	16	2.2
Employment status	Employed	287	38.8
	Herder	28	3.8
	Pensioner	155	21
	Student	201	27.2
	Unemployed	68	9.2
Income per month	<USD175	500	67.7
	USD175–525	231	31.3
	>USD525	8	1.1

* re-married, co-habiting, separated, divorced, and widowed.

**Table 2:** Response distribution for each BOS item (*n* = 739).

Item	Response Category (%) *				
	1	2	3	4	5
**Domain 1: Excessive thinking**					
I tended to overthink even the (most) minor of events.	21.8	41.8	19.9	12.3	4.2
I tended to stick to one way of doing or thinking about something.	38.2	31.9	19.1	8.5	2.3
I had racing thoughts (was thinking something a lot).	15.6	42.8	21.5	14.5	5.7
**Domain 2: Hypersensitivity**					
I felt tired when reading newspapers/magazines.	52.5	24.4	15.8	5.1	2.2
I felt that others were watching me.	44.8	29.1	19.4	3.8	3.0
I easily felt tense in public.	40.3	26.5	20.8	8.4	3.9
**Domain 3: Restless behavior**					
I did not like to have leisure time.	38.6	23.5	22.5	11.2	4.2
I tended to walk or move fast.	37.1	21.2	13.9	19.9	7.8
I was not good at waiting.	38.6	23.4	14.2	13.7	10.1
My notebook was full of schedules.	40.2	25.7	19.5	11.2	3.4

BOS: Brain Overwork Scale; * 1: very inaccurate/never (0 day per week); 2: moderately inaccurate/rarely (1–2 day(s) per week); 3: neutral/sometimes (3–4 days per week); 4: moderately accurate/frequently (5–6 days per week); 5: very accurate/always (7 days per week).

**Table 3:** Means, standard deviations (SD), Cronbach’s α and McDonald’s ω of BOS items and domains (*n* = 739).

Item	Mean	SD	Cronbach’s α	McDonald’s ω
Overall reliability	2.18	0.767	0.859	0.861
I tended to overthink even the (most) minor of events	2.35	1.08	0.846	0.848
2. I tended to stick to one way of doing or thinking about something	2.05	1.06	0.845	0.846
3. I had racing thoughts (was thinking something a lot)	2.52	1.09	0.850	0.851
4. I felt tired when reading newspapers/magazines	1.80	1.02	0.849	0.850
5. I felt that others were watching me	1.91	1.03	0.847	0.848
6. I easily felt tense in public	2.09	1.14	0.846	0.847
7. I did not like to have leisure time	2.19	1.18	0.847	0.849
8. I tended to walk or move fast	2.40	1.36	0.841	0.843
9. I was not good at waiting	2.33	1.37	0.843	0.844
10. My notebook was full of schedules	2.12	1.16	0.845	0.847
Domain 1: Excessive thinking	6.92	2.59	0.818	0.908
Domain 2: Hypersensitivity	5.80	2.55	0.821	0.909
Domain 3: Restless behavior	9.04	3.94	0.742	0.889
BOS total score	21.77	7.67	0.769	0.792

**Table 4:** Intraclass correlation coefficients (ICC) of the BOS (*n* = 366).

Domains	Mean ± Standard Deviation		ICC
	Test	Retest	
Domain 1: Excessive thinking	9.11 ± 2.91	8.72 ± 2.98	0.730
Domain 2: Hypersensitivity	7.72 ± 2.66	7.49 ± 2.55	0.690
Domain 3: Restless behavior	11.8 ± 3.01	11.7 ± 2.97	0.650
BOS total score	28.7 ± 6.85	27.9 ± 7.11	0.750

**Table 5:** Principal component analysis of the BOS (*n* = 739).

Components	Factor Loadings (Explained Variance %)			Uniqueness
	Excessive Thinking (22.9)	Hypersensitivity (20.3)	Restless Behavior (20)	
I tended to overthink even the (most) minor of events.	0.766			0.332
I tended to stick to one way of doing or thinking about something.	0.727			0.329
I had racing thoughts (was thinking something a lot).	0.722			0.398
I felt tired when reading newspapers/magazines.		0.772		0.341
I felt that others were watching me.		0.727		0.362
I easily felt tense in public.		0.712		0.383
I did not like to have leisure time.			0.807	0.311
I tended to walk or move fast.			0.713	0.367
I was not good at waiting.			0.651	0.405
My notebook was full of schedules.			0.646	0.451

BOS: Brain Overwork Scale.

**Table 6:** Convergent validity of the BOS (*n* = 739).

Items/Domains	Domains		
	Excessive Thinking	Hypersensitivity	Restless Behavior
Item convergence (*r* ≥ 0.4)	3/3	3/3	4/4
Corrected item-total correlations			
**Domain 1: Excessive thinking**			
I tended to overthink even the (most) minor of events	0.799 ***	0.415 ***	0.466 ***
I tended to stick to one way of doing or thinking about something	0.789 ***	0.483 ***	0.467 ***
I had racing thoughts (was thinking something a lot)	0.770 ***	0.388 ***	0.459 ***
**Domain 2: Hypersensitivity/brain fatigue**			
I felt tired when reading newspapers/magazines	0.384 ***	0.784 ***	0.481 ***
I felt that others were watching me	0.477 ***	0.797 ***	0.476 ***
I easily felt tense in public	0.466 ***	0.836 ***	0.530 ***
**Domain 3: Restless behavior**			
I did not like to have leisure time	0.411 ***	0.492 ***	0.783 ***
I tended to walk or move fast	0.500 ***	0.531 ***	0.826 ***
I was not good at waiting	0.533 ***	0.498 ***	0.815 ***
My notebook was full of schedules	0.445 ***	0.540 ***	0.757 ***
**Inter-item correlations for domains**			
Domain 1: Excessive thinking	-	-	-
Domain 2: Hypersensitivity/brain fatigue	0.547 ***	-	-
Domain 3: Restless behavior	0.590 ***	0.615 ***	-

BOS: Brain Overwork Scale; *** *p* < 0.001; *p*-values were calculated using Spearman’s correlations.

**Table 7:** Criterion validity of the BOS (*n* = 739).

Questionnaires	BOS			
	BOS Total	Excessive Thinking	Hypersensitivity	Restless Behavior
HADS (Hospital Anxiety and Depression Scale)				
Anxiety	0.429 ***	0.481 ***	0.417 ***	0.248 ***
Depression	0.288 ***	0.292 ***	0.330 ***	0.160 ***
WHOQOL-BREF (An Abbreviated Version of the World Health Organization Quality of Life)				
Perception on quality of life	−0.086 *	−0.114 **	−0.126 ***	−0.003
Perception on general health	−0.254 ***	−0.263 ***	−0.268 ***	−0.150 ***
Physical health	−0.396 ***	−0.367 ***	−0.389 ***	−0.296 ***
Psychological health	−0.277 ***	−0.278 ***	−0.354 ***	−0.138 ***
Social relations	−0.161 ***	−0.168 ***	−0.250 ***	−0.033
Environmental health	−0.323 ***	−0.287 ***	−0.346 ***	−0.221 ***

BOS: Brain Overwork Scale, * *p* < 0.05, ** *p* < 0.01, and *** *p* < 0.001; *p*-values were calculated using Spearman’s correlations.

## Data Availability

Data sharing is not applicable.

## Supplementary Materials

The following supporting information can be downloaded at: https://www.mdpi.com/article/10.3390/healthcare11071003/s1, Figure S1: Conceptual framework of the Brain Overwork Syndrome; Figure S2: Study flowchart; Figure S3: Scatter plot between the BOS-10 total score and age by gender; Figure S4: The means and 95% confidence intervals of the BOS-10; Table S1: Descriptive statistics of the 37-item Brain Overwork Scale (BOS-37).
