# Supplementary material for: The Brain Overwork Scale: A Population-Based Cross-Sectional Study on the Psychometric Properties of a New 10-Item Scale to Assess Mental Distress in Mongolia
Source: Healthcare (Basel). 2023 Mar 31;11(7):1003. doi: 10.3390/healthcare11071003 (PMC10094685; doi:10.3390/healthcare11071003)
Supplement: Supplementary file 1 [file healthcare-11-01003-s001.zip › healthcare-2262796-supplementary.pdf]

Supplementary Materials:

Figure S1. Conceptual framework of “Brain Overwork Syndrome”

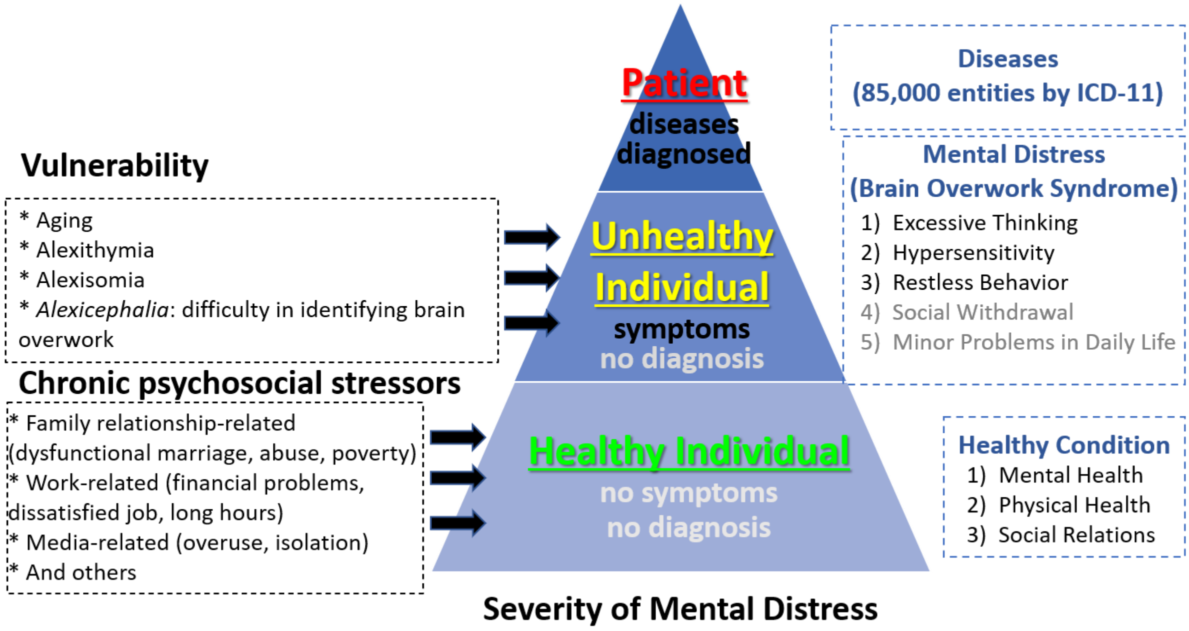

Figure S1: Conceptual frameworks “Brain Overwork Syndrome”.
Chronic psychosocial stressors cause mental distress or an abnormal condition that is characterized by the Brain Overwork Syndrome: Excessive Thinking, Hypersensitivity, Restless Behavior, Social Withdrawal, and Minor Problems in Daily Life.

Figure S2. Study flowchart

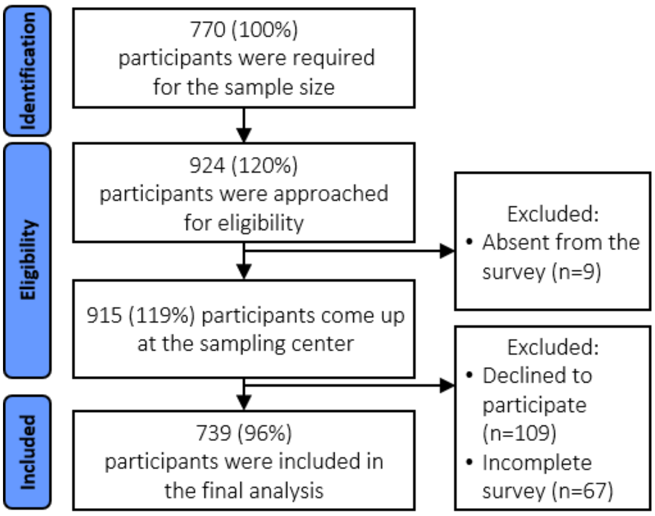

Figure S3. Scatter plot between the BOS-10 total score and age by gender

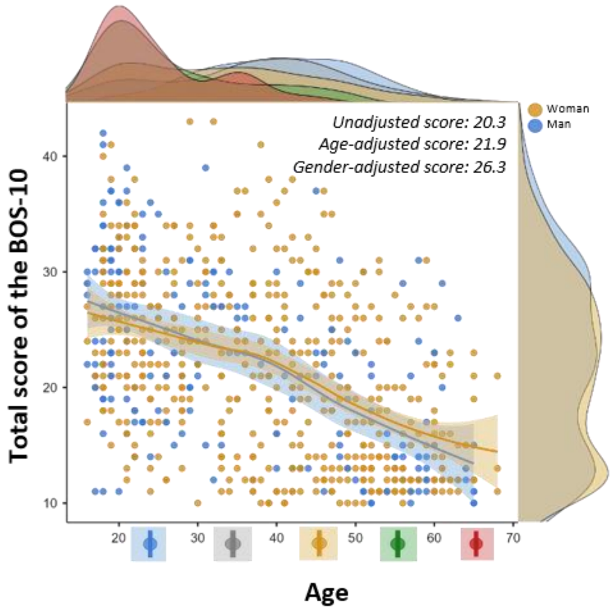

Figure S4. The means and 95% confidence intervals of the BOS-10

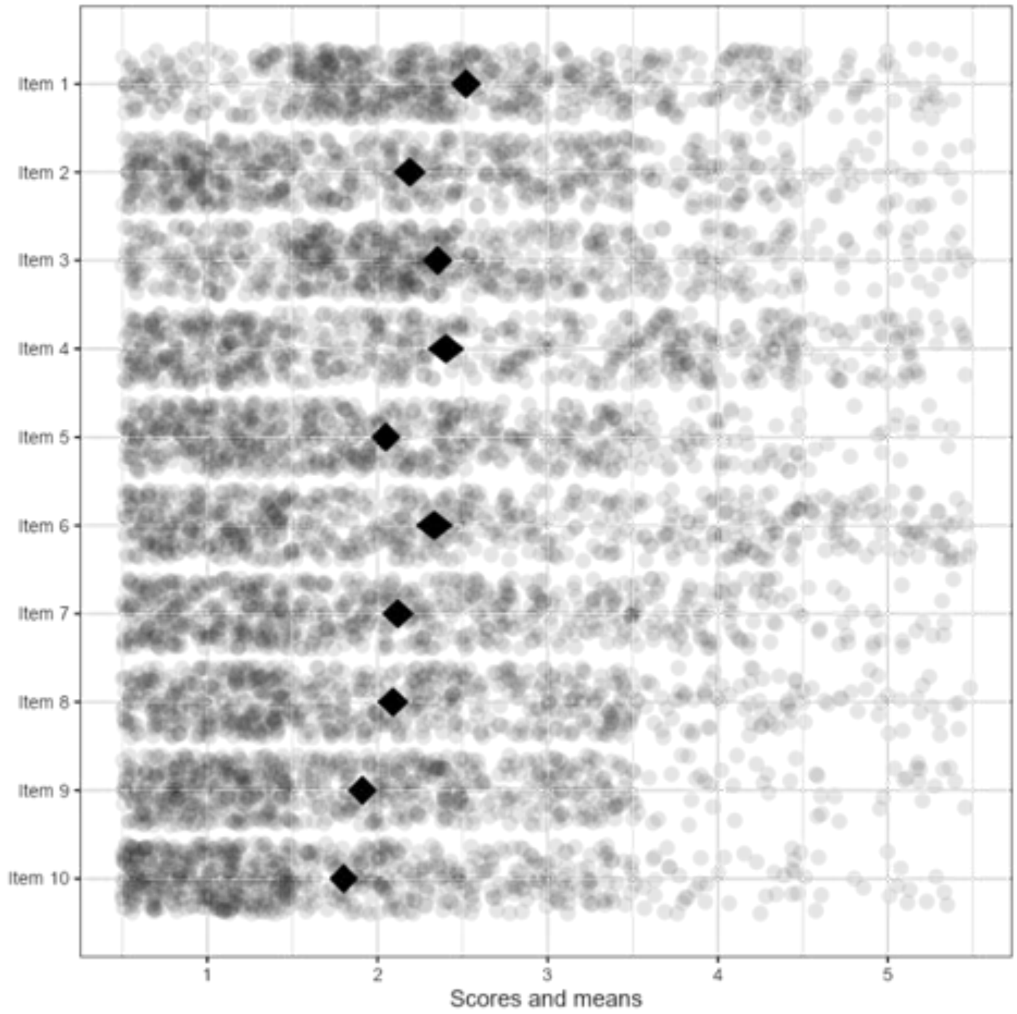

Table S1: Descriptive statistics of the 37-item Brain Overwork Scale

Please tell me about your behavior or pattern of thinking in your daily life.
Please tell me how often you have felt or behaved this way during the past week

Items	mean	SD	skewness	kurtosis
**Domain 1: Excessive thinking (ET)**				
I had racing thoughts (was thinking something a lot)	2.52	1.09	0.58	2.62
I tended to think about multiple (other) things or tasks when working	2.24	1.20	0.65	2.37
I tended to think about the past / future	2.49	1.33	0.41	1.93
I tended to overthink even the (most) minor of events	2.35	1.08	0.66	2.77
I tended to stick to one way of doing or thinking about something	2.05	1.06	0.80	2.88
**Domain 2: Hypersensitivity/brain fatigue (H)**				
I felt very sensitive to lights and/or sound	2.25	1.17	0.51	2.15
I tended to compare myself to others	1.85	0.99	1.04	3.40
I was not good at staying in a crowd	1.88	1.01	0.95	3.15
I felt tired when reading newspapers / magazines	1.80	1.02	1.18	3.68
I felt brain fog (felt my brain was not working well)	2.20	1.13	0.57	2.42
I easily felt tense in public	2.09	1.14	0.81	2.78
I felt that others were watching me	1.91	1.03	1.05	3.62
**Domain 3: Restless behavior (RB)**				
I did not like to have leisure time	2.19	1.18	0.64	2.39
I felt like I was pressed for time	2.12	1.17	0.75	2.52
I tended to walk or move fast	2.40	1.36	0.47	1.85
I was not good at waiting	2.33	1.37	0.65	2.11
I tended to rush when completing a task (e.g. household chores)	1.96	1.04	0.94	3.20
I would tell myself to not waste time	2.33	1.33	0.54	2.03
My notebook was full of schedules	2.12	1.16	0.73	2.51
I could not stay still (had such characteristic)	1.60	0.94	1.58	4.87
**Domain 4: Social withdrawal (SW)**				
I tended to refrain from asking others for help	1.91	1.05	1.02	3.29
I was not good at relying on other people	2.05	1.07	0.72	2.65
I tended to prioritize others over myself	2.08	1.14	0.69	2.38
I was not good at rejecting requests from others	2.34	1.29	0.49	1.96
I was not good at sharing my complaints with others	2.11	1.17	0.74	2.50
I tended to listen to others when talking	2.80	1.47	-0.02	1.50
**Domain 5: Minor problems in daily life (MP)**				
I had stiffness (felt heavy) in my neck / shoulders	2.75	1.16	0.30	2.15
I had heartburn	1.84	1.04	1.15	3.60
I had an upset stomach after a meal	1.78	0.90	1.10	3.94
I had night sweats	1.49	0.84	1.96	6.85
I felt tired when waking up	2.35	1.11	0.55	2.54
I felt hot headed	1.69	0.97	1.41	4.40
I had a headache	2.62	1.14	0.21	2.10
**Control questions (reverse questions)**				
I had enough time to relax	3.32	1.04	-0.50	2.40
I had enough time to stay healthy	2.94	0.99	0.08	2.59
I slept well	3.10	1.22	-0.30	2.03
I regularly exercised	2.11	1.10	0.87	3.04

BOS: Brain Overwork Scale; 1: very inaccurate / never (0 day per week), 2: moderately inaccurate / rarely (1-2 day(s) per week), 3: neutral / Sometimes (3-4 days per week), 4: moderately accurate / frequently (5-6 days per week), 5: very accurate / always (7 days per week).
